# Decadal-Scale Changes of Dinoflagellates and Diatoms in the Anomalous Baltic Sea Spring Bloom

**DOI:** 10.1371/journal.pone.0021567

**Published:** 2011-06-29

**Authors:** Riina Klais, Timo Tamminen, Anke Kremp, Kristian Spilling, Kalle Olli

**Affiliations:** 1 Institute of Ecology and Earth Sciences, University of Tartu, Tartu, Estonia; 2 Marine Research Centre, Finnish Environment Institute, Helsinki, Finland; Biodiversity Insitute of Ontario - University of Guelph, Canada

## Abstract

The algal spring bloom in the Baltic Sea represents an anomaly from the winter-spring bloom patterns worldwide in terms of frequent and recurring dominance of dinoflagellates over diatoms. Analysis of approximately 3500 spring bloom samples from the Baltic Sea monitoring programs revealed (i) that within the major basins the proportion of dinoflagellates varied from 0.1 (Kattegat) to >0.8 (central Baltic Proper), and (ii) substantial shifts (e.g. from 0.2 to 0.6 in the Gulf of Finland) in the dinoflagellate proportion over four decades. During a recent decade (1995–2004) the proportion of dinoflagellates increased relative to diatoms mostly in the northernmost basins (Gulf of Bothnia, from 0.1 to 0.4) and in the Gulf of Finland, (0.4 to 0.6) which are typically ice-covered areas. We hypothesize that in coastal areas a specific sequence of seasonal events, involving wintertime mixing and resuspension of benthic cysts, followed by proliferation in stratified thin layers under melting ice, favors successful seeding and accumulation of dense dinoflagellate populations over diatoms. This head-start of dinoflagellates by the onset of the spring bloom is decisive for successful competition with the faster growing diatoms. Massive cyst formation and spreading of cyst beds fuel the expanding and ever larger dinoflagellate blooms in the relatively shallow coastal waters. Shifts in the dominant spring bloom algal groups can have significant effects on major elemental fluxes and functioning of the Baltic Sea ecosystem, but also in the vast shelves and estuaries at high latitudes, where ice-associated cold-water dinoflagellates successfully compete with diatoms.

## Introduction

On a geological time scale, the development of ocean and atmosphere chemistry has been highly integrated with the evolution of photosynthesis in the ocean, and still today, approximately half the global C-fixation takes place in the sea [1]. This production is mainly carried out in the free water masses by phytoplankton, which is a highly heterogeneous group of microscopic algae. The unique characteristics of different groups and species of phytoplankton have over the last decades been shown to have far-reaching implications for the environment. The phytoplankton community composition may directly affect higher trophic levels of the food web [2], ocean chemistry [1] and the atmosphere, e.g. cloud albedo [3], and ocean productivity plays an integral part of global biogeochemical feedback mechanisms. Two of the most dominating phytoplankton groups: diatoms and dinoflagellates, together changed the global oceanic biogeochemistry soon after their rise, ca. 250 Myr ago [4], and in the contemporary oceans they contribute a major part of the primary production.

These two phylogenetic groups exhibit unique and distinct, often contrasting adaptive ecologies, explaining their global niche partitioning on the turbulence-nutrient matrix of habitats and onshore-offshore gradient [5]. In the temperate zone, the successional cycle in coastal waters classically begins with a winter-spring diatom bloom that is seasonally replaced by summer communities dominated by dinoflagellates [6]. Diatom blooms are of high species diversity, and a species succession generally occurs [7], [8]. Dinoflagellate blooms, in contrast, have low species diversity, and exhibit a rudimentary species succession, if any [9].

The Baltic Sea is an exceptional coastal, brackish water body, which functionally is much like a large estuary with both horizontal and vertical salinity gradients. Due to the partly enclosed geography and high anthropogenic influence, environmental problems such as eutrophication are amplified in the Baltic Sea. Environmental pressures related to eutrophication are getting more common in coastal areas worldwide (e.g. [10]), resulting in deteriorated ecosystem services and altered biogeochemical functioning of coastal zones [11]. In addition to problems with eutrophication, the Baltic Sea ecosystem is sensitive to climate change, mainly because it is greatly affected by freshwater runoff, predicted to increase in Northern Europe within decades, and by saltwater intrusions from the North Sea, which are forced by meteorological conditions [12].

The Baltic Sea is an exception to the general trend of diatom dominance during spring with a unique and anomalous niche overlap of diatoms and dinoflagellates during the spring bloom. Large (20–30 µm) cold-water dinoflagellates match or even clearly exceed the biomass of diatoms during spring bloom [13], [14] in parts of the Baltic Sea.

Despite their growth and nutrient uptake capabilities describing r- and K-strategies (diatoms and dinoflagellates, respectively), the two phylogenetic groups appear to be functional surrogates, as both are separately capable of exhausting the wintertime inorganic nutrient pools in spring, and of producing bloom-level biomasses [13]. Diatoms and dinoflagellates have basically comparable nutrient requirements (excluding the need for silica), and in the Baltic Sea, both appear to provide similar ecosystem services with respect to annual new production and nutrient uptake [15].

Several authors have suggested that the role of dinoflagellates in the Baltic Sea spring bloom has increased over the last decades, both in the northern Baltic Sea [15], [16], [17], as well as in the central and southern parts [18], [19]. Shift towards dinoflagellate dominance has mostly been linked to climate variability and changes in the physical environment, since nutrients are not limiting at the beginning of the spring bloom [15].

We hypothesized that in relation to ongoing eutrophication in the Baltic Sea, we should see the increase in both, the bloom magnitude and share of the faster growing diatoms in the spring bloom biomass. In contrast, dinoflagellates should be favored by climatic and weather conditions enhancing the water column stratification at the onset of the bloom, with distinct north-south gradient (ice cover, importance of freshwater runoff), as well as by topographical properties of sub-basins (proximity to the seed banks). If proved correct, these general hypotheses would predict different trajectories for the diatoms and dinoflagellates in different Baltic Sea sub-basins, especially under climate change.

In the present study we analyze decadal-scale trends in the Baltic Sea spring bloom dinoflagellates and diatoms. We specifically asked the following questions: (a) have the proportions of spring bloom diatoms and dinoflagellates changed on a decadal time scale; (b) are there spatial differences in the biomass, relative proportion, or rate of temporal change of either group over the Baltic Sea; (c) is the intensity of spring bloom associated with the dominance of either group; and (d) is there an association between the proportion of dinoflagellates and winter-spring climate variability, reflected by the North Atlantic Oscillation (NAO) index?

## Results

### Long-term temporal trends reveal pronounced changes in the spring bloom community structure

**Figure 1:**
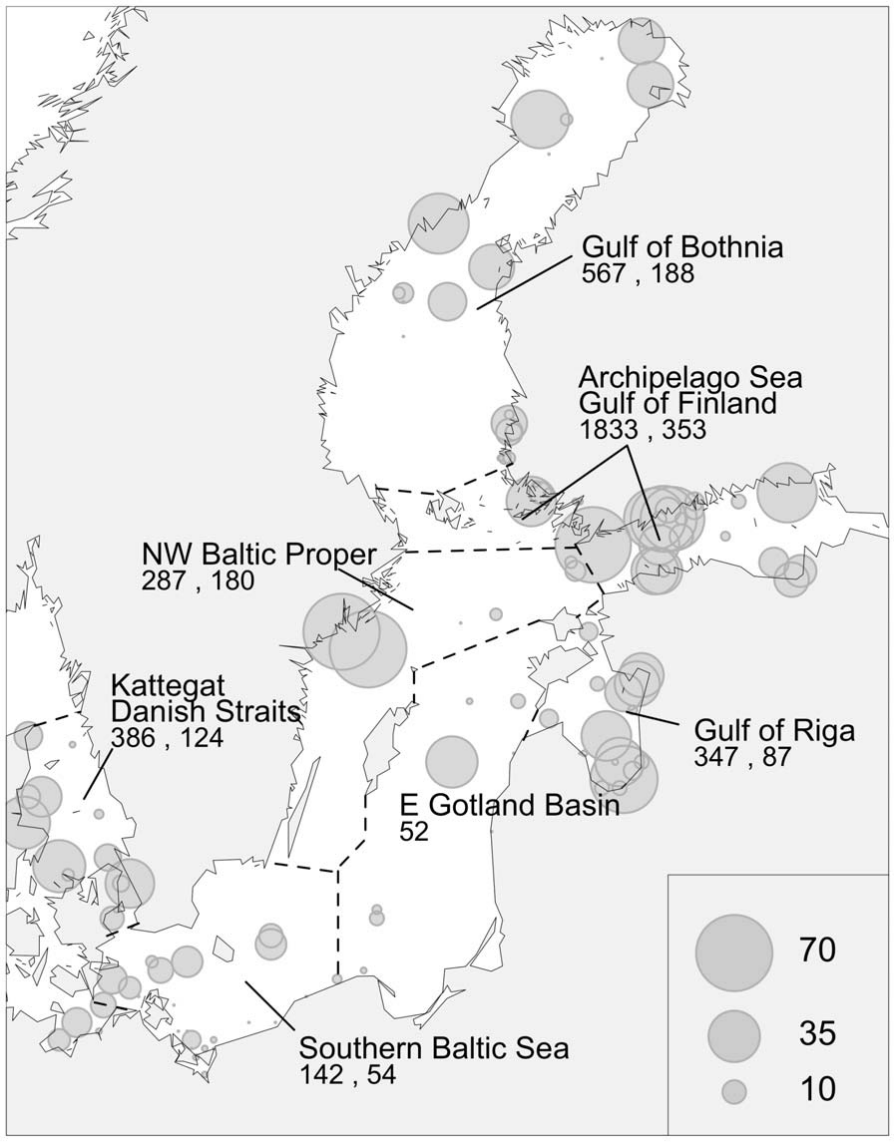
Spatial distribution of the data. Gray symbols mark the sampled stations with the symbol area proportional to the number of samples. Lines and polygons indicate the regions pooled together for long-term analyses. The numbers represent the total amount of spring bloom samples, and the number of basin-wide weakly means used for long-term analysis.

**Figure 2:**
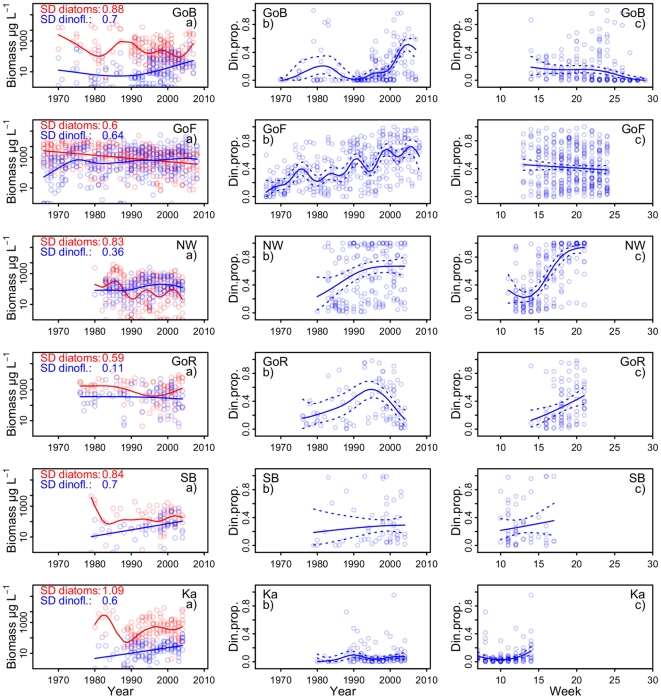
Long-term trends of diatoms and dinoflagellates in the Baltic Sea. Lines depict the long-term trends of dinoflagellate and diatom biomass (column a), dinoflagellate proportion (column b) and seasonal pattern of dinoflagellate proportion (column c) in six major basins of the Baltic Sea; GoB – Gulf of Bothnia; GoF – Gulf of Finland and Archipelago Sea; NW – northwestern Baltic Proper; GoR – Gulf of Riga; SB – Southern Baltic Sea; Ka – Kattegat, the Belt Sea and the Sound. Symbols represent basin wide weekly averages; trend lines are predicted by GAM smoothers. Eastern Gotland Basin is not shown due to poor data coverage.

The biomass of diatoms and dinoflagellates in the major basins (as defined in Fig. 1) of the Baltic Sea spring bloom has changed considerably over the decadal time period (Fig. 2). The spring blooms in the major basins of the Baltic Sea have shifted towards lower diatom and higher dinoflagellate biomass. We used standard deviations of the predicted annual values from the GAM models to compare which of the two groups revealed higher long-term biomass variability. In the Gulf of Finland, the biomass of both groups varied with almost equal amplitude over time, while in the other basins diatoms were the more variable component of the spring bloom (Fig. 2 a panels).

The long-term dynamics of the proportion of dinoflagellates had a different pattern (Fig. 2 b panels). The proportion of dinoflagellates has increased considerably in the northern Baltic Sea: the Gulf of Bothnia, the Gulf of Finland and the north and northwestern Baltic Proper. The semi-enclosed Gulf of Riga has normally diatom-dominated spring blooms, but had a high proportion of dinoflagellates in mid 1990s due to the decline of diatoms (see Discussion). The Southern Baltic Sea and the Kattegat have strongly diatom-dominated spring blooms.

In the predominantly ice-covered Gulf of Bothnia and Gulf of Finland, proportion of dinoflagellates and diatoms showed no consistent pattern through the spring bloom, in contrast to the ice-free central and southern basins, where seasonal succession of the spring bloom typically starts with a diatom dominance followed by increase of dinoflagellates (Fig. 2 c panels).

Significant positive association between dinoflagellate dominance and NAO index was found for the northwestern Baltic Proper and the Gulf of Finland (Table 1). The relationship weakened and was not significant in the basins further to the south (Gulf of Riga, Southern Baltic Proper, Kattegat) (Table 1). The model intercept parameter (0.54, variance 0.08), when back-transformed, sine(0.54)^2^, suggests that the overall proportion of dinoflagellates at zero NAO index was ca. 0.26. The average dinoflagellate proportion in the Baltic Sea spring blooms has exceeded 0.26, which is in line with the predominantly positive NAO phase during the last decades in the region.

**Table 1:** Effect of NAO on dinoflagellate proportions in six major basins.

Basin	Parameter estimate	Standard error	p-value
Gulf of Bothnia	−0.04	0.04	0.28
Gulf of Finland	0.08	0.03	0.003
Northwestern Baltic Proper	0.11	0.04	0.01
Gulf of Riga	0.07	0.05	0.16
Southern Baltic Sea	0.08	0.06	0.14
Kattegat and Danish straits	−0.02	0.04	0.56

Basin-specific slope parameters (dinoflagellate proportion vs NAO index) from the mixed effects model with estimated standard errors and p-values. The parameter values quantify the change of the arcsin-square root transformed dinoflagellate proportion (range 0 to 1.5) per unit change of the NAO index.

We were interested in the direction and strength of the effect of the NAO index in different basins (the interaction term) and not so much the main effect of basins. Therefore basin-specific intercepts were not calculated; basins were treated as a random component in the mixed effect statistical model, reducing the number of estimated parameters and preserving the degrees of freedom.

### Spatial variability in the spring bloom biomass, the proportion of dinoflagellates, and rate of change of dinoflagellate dominance

**Figure 3:**
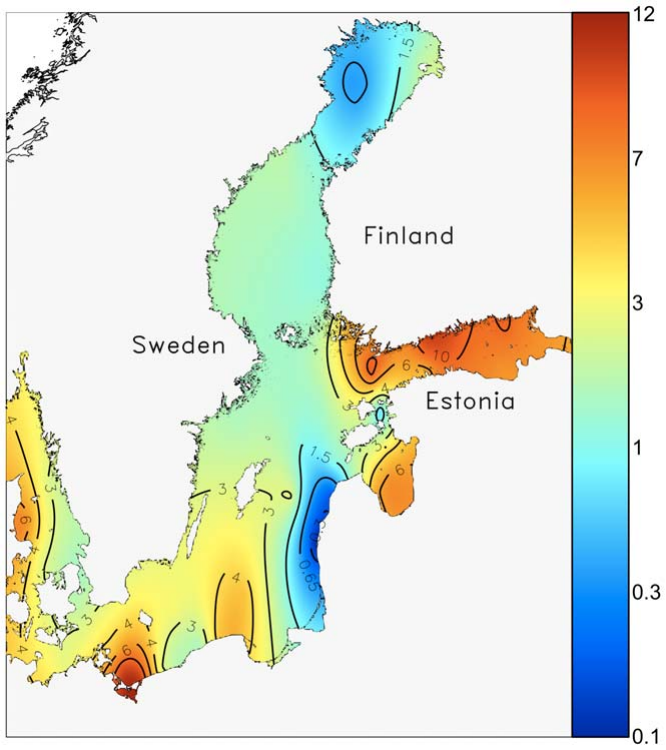
Spatial variability of the spring bloom peak biomass (wet weight, mg L^−1^) in the Baltic Sea. High biomass is characteristic to the eutrophied basins (Gulf of Finland, Gulf of Riga), with a biomass >6 mg L^−1^. For most of the Baltic Sea, peak biomass of the spring bloom samples falls between 1.5–3 mg L^−1^.

The spatial pattern of the average total spring bloom biomass (Fig. 3) reflects the general knowledge of the Baltic Sea eutrophication due to local nutrient loading [20], with the Gulf of Finland and the Gulf of Riga showing the highest spring bloom biomass values (>6 mg L^−1^).

**Figure 4:**
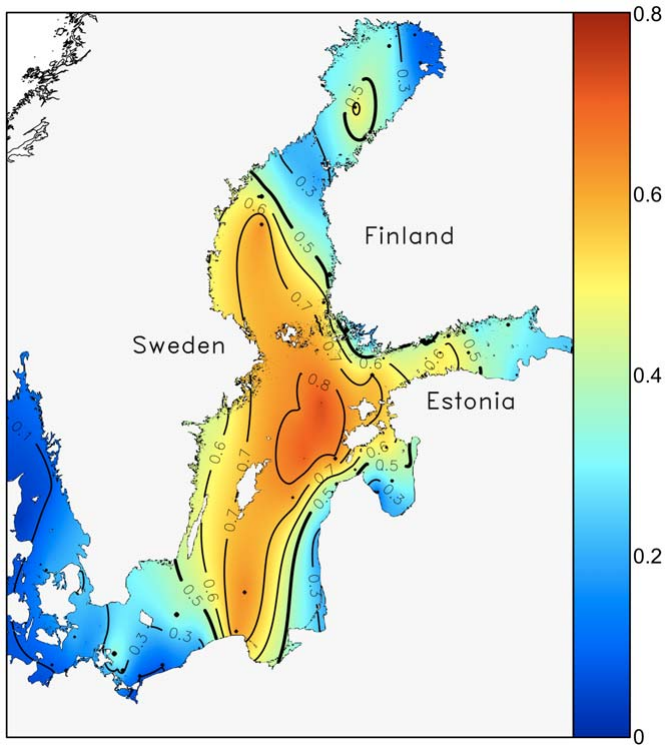
Spatial variability in the proportion of dinoflagellates interpolated with ordinary kriging. The thick contour line separates dinoflagellate dominance (>0.5) and diatom dominance (<0.5).

There was apparently no association between the spring bloom biomass and dominance patterns of dinoflagellates or diatoms (Fig. 4), as diatoms hold a strong position both in the oligotrophic northern basins (Bothnian Bay) as well as in eutrophied Gulf of Riga, Kattegat, Danish coastal waters and in the Southern Baltic Sea, where the spring bloom biomass is relatively high (3–4 mg L^−1^), particularly in the Bay of Kiel (ca. 6 mg L^−1^). Similarly, dinoflagellates dominate the spring blooms both in the highly eutrophied Gulf of Finland as well as in the offshore waters of the northern Baltic Proper (>0.8), which shows moderate to low average spring bloom biomass values (ca. 1.5 mg L^−1^).

**Figure 5:**
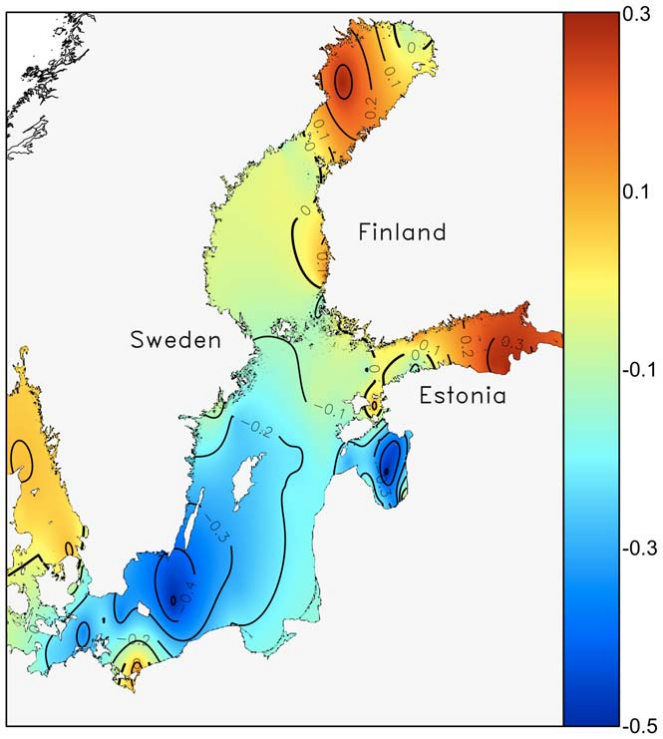
Shifts in the proportion of dinoflagellates over the period of ten years (1995 to 2004). The predictions were made by geographically weighted linear regression and interpolated with ordinary kriging. Positive and negative values represent the areas of increasing and decreasing dinoflagellate proportion, respectively. Thick contour lines denote boundary between areas of increasing and decreasing trend.

Figure 5 shows the spatial patterns of the direction and average linear rate of the change of dinoflagellate proportion between 1995 and 2004. During this decade, dinoflagellates have increased in most of the northern and eastern parts of the Baltic Sea, particularly in the Gulf of Bothnia and the Gulf of Finland. Notably, the rate of change is slightly negative in the deep offshore areas of the central and northern Baltic Proper, where the average proportion of dinoflagellates was particularly high (>0.8) during 1995–2004 (Fig. 4).

## Discussion

The proportion of diatoms and dinoflagellates in the Baltic Sea spring bloom has been highly variable in time and space during the past 4 decades. No common all-Baltic trend or pattern can be shown for the different areas of the Sea, indicating that mechanisms driving the change have a strong regional and fine-scaled component, characteristic for coastal ecosystems in general [21].

The most pronounced decadal-scale increase in the dinoflagellate proportion from 0.1 to 0.7 has taken place in the eutrophied Gulf of Finland. Dinoflagellates have also increased in the Bothnian Bay and the Bothnian Sea, but spring blooms in these northern basins are still largely diatom-dominated. In the central, offshore Baltic Sea, where the spring bloom biomass is relatively low, dinoflagellates hold a strong, dominating position (>0.7). The main basins where diatoms dominate the spring bloom are the northern Bothnian Bay, the southwestern Baltic Sea and also the eutrophied Gulf of Riga, but in all these basins, long-term, temporal changes have been distinct (Fig. 2).

No consistent association between the relative dominance of diatoms or dinoflagellates, and the intensity of the spring bloom, was found across the sub-basins. This supports the functionally surrogate role of these phylogenetic groups in system-wide biogeochemical cycles and undermines the hypothesis that anthropogenic nutrient enrichment favors the fast-growing diatoms.

### Does dissolved silicate decrease drive dinoflagellate expansion in the Baltic Sea?

Decreasing dissolved silica (DSi) availability and lowering DSi:N ratios, associated with eutrophication [22], [23] and decreasing riverine DSi inputs [24], have been suggested to limit diatom growth in the Baltic Sea, and thus indirectly support the expansion of dinoflagellate blooms. However, as shown recently, the most common Baltic spring diatoms are well adapted to low DSi concentrations and not directly affected by decreased surface levels [25]. DSi concentrations are sufficient during the early phase of the spring bloom when the competition determining the composition at the bloom peak is largely decided [15].

However, some spring bloom diatoms in the Baltic Sea rely entirely on benthic resting stages to survive the warm summer, and require large amounts of DSi when heavily silicified spores are formed [26]. At the late phase of the bloom, low DSi may thus lead to poor sporulation and subsequently compromise the seeding and competitive success of the next generation of these diatoms. Late-bloom DSi depletion is also likely to affect the competition between different diatom species, as the degree of silicification varies significantly between Baltic Sea bloom-forming diatoms [25].

In specific cases, low DSi may set the upper limit of diatom spring bloom magnitude. Long-term data analysis showed that the semi-enclosed Gulf of Riga went through a DSi depletion event culminating in 1993 [27], coinciding with the significant increase of dinoflagellate abundance (Fig. 2). The likely cause was a complex interplay between exceptional blooms of heavily silicified diatom species, and slow dissolution of biogenic silica resulting in drawdown of DSi stocks for several years. Since 1995, the DSi concentrations and the proportion of diatoms in the Gulf of Riga have recovered, leading to rapidly decreasing dinoflagellate proportion.

### Habitat specialists vs. generalists

Globally, diatoms are ubiquitous generalists thriving in most aquatic habitats, while habitat specialization of dinoflagellates is considered to make them particularly responsive to niche changes. Altered niche structure and community ecology leads to geographic range expansion, newly achieved competitive dominance, and bloom expansion of dinoflagellates [5], [9]. However, the conditions, factors and mechanisms selecting for the phylogenetic group, genus, and species for bloom time windows remain poorly understood especially in variable coastal environments, and the outcome is thus highly unpredictable.

The spring bloom dinoflagellates of the Baltic Sea are inferior growth competitors compared to diatoms when nutrients are plentiful. They also tend to have a lower light saturation level, and appear to be better competitors when nutrients are low [15], [28]. In an experimental multi-year study with mixed natural communities, Kremp et al. [15] argued that the size of the inoculums population is decisive for which phylogenetic group becomes selected. Once the head-start population had been established, nutrient levels, nutrient ratios, or light intensity had only a limited effect on the outcome of the competition or biomass distribution among functional groups [15].

Competition between cold-water diatoms and dinoflagellates can therefore be interpreted in a planktonic r vs. K-selection framework. Small fast-growing diatoms thrive in unstable, turbulent conditions (corresponding to classical r-strategy), while slow-growing, large and motile dinoflagellates seem to require a specific habitat setting for bloom formation, allowing them to acquire competitive advantage through building a superior head-start biomass (K-strategy).

### Stratification dynamics driving bloom habitats

Stratification of the water column is generally a prerequisite for most dinoflagellate blooms to develop in temperate areas (e.g. [29]). As the coastal planktonic habitat fluctuates interannually and within the spring season, success strategy shifts between the habitat specialist, monospecific dinoflagellate blooms, and the high-diversity, generalist diatom blooms [9].

In the Baltic Sea, dinoflagellates are more sensitive to hydrographic conditions and climate fluctuations than to the nutrient conditions at the bloom onset. It has been shown that initial stratification, necessary for spring bloom commencement, is due to spreading of freshwater from the coastal zone in the Baltic Sea, instead of the temperature-driven stratification characteristic of lakes and full-saline marine regions [30], indicating also different bloom start situations for coast-near and offshore regions of the Baltic Sea.

Early salinity stratification is found in biotopes with large freshwater inflow, such as estuaries, and in the literature, there are a few examples of dinoflagellate-dominated estuarine spring blooms, e.g. in the uppermost Chesapeake Bay [31], and in parts of the Neuse River Estuary [32]. However, dinoflagellate dominance during spring bloom in estuaries is not a general phenomenon, as for example strong tidal mixing favors diatoms over dinoflagellates [33], and hence stratification cannot be the sole reason for the dinoflagellate dominance during spring bloom in the Baltic Sea.

### Life-history strategies of Baltic spring dinoflagellates promote bloom expansion

The life-cycle strategy of the dominant dinoflagellate species in the Gulf of Finland and the northern Baltic Sea, *Biecheleria baltica* involves massive cyst formation at the end of the bloom. Extensive cyst beds formed in the Gulf of Finland [17], [34], [35] are gradually growing and spreading with bottom water transport in the benthic nepheloid layer. These processes fuel the expanding and ever larger dinoflagellate blooms in the relatively shallow basin of the Gulf of Finland. The close proximity to the cyst beds is likely to promote a successful return of the species to surface waters here. Notably, *B. baltica* has negligible bloom intensity in the nearby Gulf of Riga, where cysts of this species are virtually absent from the sediment [17].

The strategy of *B. baltica* to produce such large quantities of survival stages is relatively uncommon among the better known dinoflagellate species. Cyst yields of most dinoflagellates studied in this respect, are significantly lower than of *B. baltica*, with cyst fluxes and cyst concentrations in the sediment differing by orders of magnitudes [17], [36]. We assume that in an environment that allows successful re-introduction of seeds to the euphotic layer, the strategy of excessive “seed” formation makes *B. baltica* more competitive with diatoms than other dinoflagellates.

Large inocula of seeds at the beginning of the growth season may compensate for the slow growth of the dinoflagellates and enable them to persist against diatoms, which, due to their high growth rates, are generally very effectively seeded even from minor inocula of resting propagules [37]. In the northern Baltic Sea, resting propagules of all common spring diatoms are abundant in coastal sediments and likely to be seeded successfully (Kremp, unpublished data) when mixed into the water column. In the open Baltic Proper, the main spring bloom dinoflagellate, a recently described *Gymnodinium corollarium*, produces also conspicuous quantities of resting cysts [38].

### A habitat succession hypothesis for the Baltic Sea dinoflagellate expansion

Specific hydrological settings and a seasonal sequence of events are required for the dominance of cold-water dinoflagellates during the spring bloom in coastal waters. Wintertime mixing incidents promote introduction of benthic seed populations to the upper water column [39]. As also diatoms deposit resting stages to sediments, the mixing events are necessary, but not sufficient for dinoflagellate dominance. A consequent period of water column stability, particularly under the ice cover, disfavors competing diatoms, which are prone to sedimentation, while irradiance is still insufficient for bloom development. This concomitantly favors shade-adapted motile dinoflagellates and promotes the build-up of high cell densities. These dense populations in thin surface layers provide the necessary head-start by the onset of the spring bloom, which is decisive for the dominance of dinoflagellates during the peak of the spring bloom, confounding the conventional sequence from diatoms to dinoflagellates [15], [40].

The conspicuous dominance of dinoflagellates in the ice-free central Baltic Proper calls for further research. We believe the permanent halocline at 60–80 m depth in the Gotland Basin is a strong barrier for local cyst re-suspension from the sediment surface to the euphotic layer. Anoxic sediments and bottom waters in the deep parts of the Gotland Basin are not favorable for cyst germination [41], [42], but when re-suspended to oxic conditions and germinating, the motility of dinoflagellates is a major advantage over the non-motile diatoms to cross the salinity stratification and reach the surface layer. We are not aware of comparable investigations from the Baltic Sea, but modeling studies from the Gulf of Maine (US east coast) suggest that germinating cells from the offshore dense cyst beds at 150 m and deeper largely determine the timing and intensity of the dinoflagellate blooms, supported by their average upwards swimming speed of 5–15 m day^−1^ [43], [44].

The observed differences in bloom dominance between Baltic Sea sub-basins can thus be assumed to reflect physical habitat properties: topographical differences (proximity to sediment seed banks), distance to major freshwater discharges promoting initial stratification, and the N-S gradient governing the probability and extent of ice cover, and the permanent salinity stratification in deeper off-shore areas.

It remains open, however, exactly how these physical differences relate to the different dominance patterns and trends in different sub-basins. If ice cover, freshwater-induced stratification, and relatively shallow seed banks are instrumental for the observed *Biecheleria baltica* success in the Gulf of Finland, why does the species not thrive in the Gulf of Bothnia, where these habitat properties play even a stronger role? Instead, the recent increase in dinoflagellate proportion in both the latter Gulf and the deep, usually ice-free Baltic proper is due to another effective cyst former, *Gymnodinium corollarium* while the Gulf of Riga, which witnessed strongly increasing dinoflagellate proportions in 1990's, appears to be a domain of *Peridiniella catenata*.

A combination of a founder effect, low connectivity between basins, and a threshold in cyst accumulation in sediments, before a hang-around species is able to build the necessary head-start under supportive physical conditions, and thus to secure future success in the habitat, seems a plausible hypothesis for the demonstrated dinoflagellate expansion, but it cannot be verified by the current data. Coupling of data on species-specific life cycles and succession to physical circulation and mixing models, as well as to ice cover dynamics, appears necessary to explain the different outcomes in different neighboring habitats. The overall picture anyhow emphasizes the habitat specialist character of cold-water dinoflagellates, as previously addressed to their warm-water relatives.

### Can climate variability influence the spring bloom composition?

Inter-annual variability in weather conditions, e.g. mild or harsh winters, the ice cover and its extent, and the sea surface temperature have frequently been suggested to shape the vernal phytoplankton assemblage in the different sub-regions of the Baltic Sea [13], [14]. Our data indicate that in the northwestern Baltic Proper and in the Gulf of Finland, the phase of NAO is linked to the spring bloom species composition and the relative proportions of diatoms and dinoflagellates. However, in most of the Baltic Sea basins the relation between dinoflagellate proportion and NAO index is weak, lacking, or even opposite (e.g. the Gulf of Bothnia), suggesting that other factors, not accounted for here, have been important.

In the predominantly ice-free northwestern Baltic Proper, the significant effect of NAO could be explained by the strong seasonal sequence of the spring blooms, starting with diatoms and proceeding with dinoflagellates. Our preliminary analysis (data not shown) suggests that the successional shift from diatoms to dinoflagellates takes place earlier in the season in years of positive NAO phase, leading to significantly higher overall dominance of dinoflagellates. Increasing dinoflagellate proportion towards the end of the spring bloom appears to be characteristic for all ice-free central and southern areas of the Baltic Sea. Yet higher dinoflagellate proportions do not appear to link specifically with positive NAO phase anywhere else than in the Gulf of Finland and northwestern Baltic Proper.

In areas with regular ice-cover (the Gulfs of Bothnia and Finland), development of stratification is less predictable and therefore less connected to a climate proxy as coarse as the winter NAO index. The local extent and the type of the ice-cover seem to pose the highest uncertainty on the seeding success of the spring blooms, and require in-depth analysis.

### Spring bloom composition affects biogeochemical cycles

Shifts in the spring bloom composition have consequences on the biogeochemical cycling and fate of the new production. In the northern temperate and boreal seas, including the Baltic Sea, the spring bloom typically dominates the annual phytoplankton productivity cycle. The spring bloom lasts approximately 1 month, but contributes 40 to 60% of the annual carbon fixation, and due to the mismatch between the timing of spring bloom and development of main grazers in the Baltic Sea, up to 80% of this fixed carbon sinks out of the euphotic layer, feeding the benthic system [13], [45].

The relative abundance of diatoms or dinoflagellates, or the dominance of either group determines the proportion and quality of the new production that settles to the sea floor, and how much is disintegrated in the upper water column [13]. Diatom-dominated spring blooms terminate with a conspicuous flux of fresh organic matter and intact cells sink out of the pelagic zone and into the sediments [46]. In contrast, dinoflagellate-dominated spring blooms either disintegrate in the upper mixed layer and fuel the microbial food-web while retaining the nutrients in the productive layer [47], or go through sexual reproduction and encystment, producing cysts that are resistant to degradation in the sediment.

Spring bloom dinoflagellates can deposit up to 3×10^11^ cysts m^−2^, equivalent to 16 g C m^−2^, during a strong bloom [17], [35], which is a substantial fraction of the annual organic carbon flux in the region (30 to 50 g C m^−2^). Spilling & Lindström [48] demonstrated that when most of the settling material consisted of dinoflagellate resting cysts, oxygen consumption at the sediment surface was notably lower compared to material consisting of vegetative cells of diatoms or dinoflagellates, suggesting that biomass in the form of cysts is not readily available to the benthic decomposers.

Benthic decomposition rates directly affect basin-wide eutrophication processes through associated oxygen decomposition and phosphorus release. The northern Baltic Sea spring blooms are nitrogen-limited, while summertime planktonic communities display spatially extensive N_2_-fixing cyanobacterial blooms, promoted by the benthic P release [49], [50]. These intertwined biogeochemical cycles can boost system eutrophication into a self-enforcing vicious cycle, with increased spring biomass sedimentation as the driver [49]. Because the composition of the spring bloom has a direct impact on benthic processes, a shift to more cyst-producing dinoflagellates could potentially dampen internal nutrient loading and consequently alleviate eutrophication processe.

### Global perspectives

Much of the discussion on dinoflagellates as habitat specialists and global expansion focuses on warm-water 'red tide' species, which are temporally and often spatially distinct from the spring bloom, or upwelling-induced diatom blooms [5],[6]. The mainstream ecological literature has largely ignored the role and widespread occurrence of cold-water dinoflagellates, which co-occur and successfully compete with spring bloom diatoms. Such habitats are found along the vast Eurasian Arctic shelves, which host the ice-associated *Peridiniella catenata* [51], one of the major spring bloom dinoflagellates also in the Baltic Sea. Cold-water dinoflagellates are apparently abundant in the Arctic estuaries and river plumes [52], [53], but also in Antarctic saline lakes [54] and sea ice [55].

This ignorance is unfortunate, since climatic forcing is most accentuated in the polar regions, projected to cause future changes in habitats over large oceanic and shelf areas. In all these regions, early stratification is induced by salinity, either due to ice melt or river plumes, rather than by temperature. This feature, together with the relatively shallow depths enabling the formation of extensive benthic cyst beds, seem to be the key features associated with success of dinoflagellates over diatoms, in the Baltic Sea as well as worldwide.

In the era of rapid environmental changes in the polar regions, either due to climate change or direct anthropogenic pressure, we can expect cold-water bloom-forming dinoflagellates to successfully exploit the newly opened niches, leading to their increased role in the coastal carbon cycles, as has taken place in the Baltic Sea during the past decades. The interplay between local hydrography, climatic and weather forcing, and the adaptive strategies of species are an obvious source of this variability [21], which remains a challenge for prognostic coastal spring bloom models.

## Materials and Methods

The spatial and temporal distribution of phytoplankton was obtained from monitoring datasets provided by national monitoring agencies around the Baltic Sea (see Acknowledgements). The original data tables from national agencies or international organizations (HELCOM) were structurally unified and the taxonomy was carefully harmonized. With a few exceptions, the phytoplankton data were counted from Lugol fixed, surface mixed layer integrated samples (0–5 or 0–10 m) with an inverted microscope after settling for 24 h as suggested by Edler [56]. The spring bloom species in the Baltic Sea do not form conspicuous sub-surface maxima, as the seasonal stratification only starts to evolve during the bloom period, and we believe that the surface mixed layer gives an unbiased representation of the proportion and volumetric biomass of dinoflagellates and diatom populations. Species-specific cell volumes were used to calculate the wet weight biomass [56]. No semi-quantitative samples were used.

The onset and duration of the spring bloom varies with latitude in the Baltic Sea. Based on seasonal development of the total phytoplankton biomass we used the following basin-specific time windows: day of the year 100–200 in the Bothnian Bay, 100–180 in the Bothnian Sea and Quark, 80–160 in the Northern Baltic Proper and Archipelago Sea, 100–170 in the Gulf of Finland, 80–160 in the Eastern Gotland Basin and the Gulf of Riga, 60–120 in the Southern Baltic Proper (incl. Bornholm basin) and 50–100 in the Sound, Belt Sea and Kattegat. Altogether 3500 quantitative spring blooms samples were used in the study.

All statistics were done in the R computing environment [57], with specific analysis relying on libraries detailed below.

### Long-term trends and seasonal patterns of diatoms and dinoflagellates

The long-term trends in dinoflagellate and diatom biomass, and the proportion of dinoflagellates (the biomass of dinoflagellates divided by the combined biomass of diatoms and dinoflagellates), were analyzed for the entire temporal time span according to the basin specific availability of data. In order to show the most general trends, we pooled the data into six datasets after the major basins: Gulf of Bothnia (Bothnian Sea and Bothnian Bay), Gulf of Finland and the Archipelago Sea, northwestern Baltic Proper, Gulf of Riga, Southern Baltic Sea, and Kattegat with the straits of Denmark (the Sound and the Belt Sea) (Fig. 1). To account for the high variability of the raw biomass data, the basin-wide weekly averages of the log-transformed biomass of diatoms and dinoflagellates, and dinoflagellate proportion were used.

With no reason to assume any parametric relationship between the phytoplankton biomass variables and the time, the long-term trends (sampling year as explanatory variable) and seasonal succession patterns (day of the year as explanatory variable) were analyzed with generalized additive models (GAM). GAM is a non-parametric method that fits a smoothing curve through the data. ‘*gam’* function from *‘mgcv’* package in R [58] was chosen, as it uses cross-validation, a process that automatically determines the optimal amount of smoothing. The function uses cubic regression spline method, where the x-axis (here observation year) is divided into a certain number of intervals. In each interval, a cubic polynomial is fitted, and the fitted values per segment are linked together to form the smoothing curve [59]. When the proportion data was the response variable, we used arcsine square root transformation to normalize error distribution, and for clarity present the data in back-transformed format.

### Spatial variability in the spring bloom biomass

Phytoplankton samples from the period 1995–2004 (1500 samples) were used to estimate the spatial distribution of the spring bloom peak biomass in the Baltic Sea. For each unique sampling location (latitude, longitude) present in the dataset, the mean of the upper quartile of log-transformed biomass values was calculated based on the samples within 50 km radius.

The point estimates were then spatially interpolated using a geostatistical kriging method (*‘geoR’* software library [60]). First, an empirical exponential variogram model was fitted to the point estimates of spring bloom biomass to explore the spatial structure of autocorrelation. Next, the spatial prediction of the biomass over a regular grid of the Baltic Sea was calculated using ordinary kriging algorithm with covariance parameters estimated from the variogram model. All data points were given equal weight and the best linear, unbiased estimate at each grid location was calculated. The uneven distribution of stations leads to uncertainty in the predictions in areas with low sampling density, but the broad basin-wide distribution features are robust, as indicated by convergent results obtained by varying kriging options and variogram models.

### Spatial patterns in the average proportion and decadal change of dinoflagellates

Ordinary kriging (as in previous section) was also used to estimate the average proportion of dinoflagellates in 1995 to 2004 over the Baltic Sea grid. The proportion of dinoflagellates in all the 1500 samples was used with equal weight in the kriging algorithm.

In addition, the direction and rate of change of dinoflagellate proportion was analyzed with geographically weighted regression (GWR) (library *‘spgwr’* in R). GWR is a statistical tool for analyzing spatially varying linear relationships [61]. Briefly, global linear regression model was first calculated for the entire dataset and then calibrated for each regression point (pre-defined geographic location) with an arbitrarily chosen spatial resolution. For the latter, a geographic region was delineated around each regression point, and observations within the region were used to calibrate the global model. Based on the geographic distance between the regression point and each observation, a gaussian weighting scheme was used. Hence, observations closer to the regression point had more weight in the local regression model [61]. All unique geographic locations present in the original data (geographical coordinates of samples) were used as regression points. As a result, GWR returned local regression coefficients for each sampled point. The point estimates of regression coefficients were then spatially interpolated using an ordinary kriging method as already described in previous sections.

### Climate variability and spring bloom dinoflagellate proportion

NAO index, describing the major variability of the climatic conditions over the northern hemisphere, is frequently used as a proxy to characterize the climate-related physical forcing (temperature, precipitation, ice cover, onset of spring stratification) on the biological components of the Baltic Sea ecosystem [19]. We were interested in the association between NAO index and the proportion of dinoflagellates, and if this association differs in various Baltic Sea basins. We modeled dinoflagellate proportion as a function of NAO index in the six major basins as defined in section ‘Long-term trends and seasonal patterns of diatoms and dinoflagellates'. We used a linear mixed effects model (*‘nlme’* package in R) with NAO index–basin interaction term as a fixed effect (estimating an individual slope for each basin), and intercept per basin as a random effect. The dinoflagellate proportion was arcsine - square root transformed to normalize the error distribution. Phytoplankton field populations have high spatial and temporal variability (patchiness). Sampling these populations inevitably incorporates both, spatial and temporal autocorrelation, which are not trivial to separate with the existing statistical methods. We used basin-wide averages of dinoflagellate proportions to reduce the effect of spatial variation and autocorrelation. To account for temporal autocorrelation we used weekly averages of dinoflagellate proportions and a correlation structure based on exponential variogram model. Values of NAO index are freely available from the Climate Analysis Section, NCAR, Boulder, USA (http://www.cgd.ucar.edu/cas/jhurrell/indices.html).
